# A conversational agent as a virtual therapist for patients diagnosed with schizophrenia: A preliminary study

**DOI:** 10.1371/journal.pone.0343519

**Published:** 2026-02-27

**Authors:** Izabela Stefaniak, Karolina Gabor-Siatkowska, Marek Kozłowski, Artur Janicki

**Affiliations:** 1 Faculty of Medicine, Lazarski University, Warsaw, Poland; 2 Faculty of Electronics and Information Technology, Warsaw University of Technology, Warsaw, Poland; New York University Abu Dhabi, UNITED ARAB EMIRATES

## Abstract

**Introduction:**

Schizophrenia is a complex mental disorder characterized by disturbances in thinking, emotions, and behavior. Emotional dysregulation is a significant clinical issue. Recent years have seen growing interest in the use of conversational agents in mental health care, including for schizophrenia. This study explores the use of a conversational agent as virtual therapist (Terabot) designed to support the recognition and regulation of three emotions (anger, shame, and fear) in hospitalized individuals with schizophrenia. The pilot study aimed to assess the acceptability of this innovative intervention, focusing on protocol implementation, technical performance, and patient experience.

**Methods:**

The study employed a mixed-methods design involving 35 inpatients diagnosed with schizophrenia (ICD-10: F20.0–F20.9). Quantitative evaluation was based on the Brief Psychiatric Rating Scale (BPRS) and a structured acceptability survey, while qualitative thematic analysis was conducted on patients’ open-ended responses and facilitators’ notes. Research procedures followed COREQ standards.

**Results:**

The study involved 35 patients; 34 completed the quantitative scales, and 32 completed the acceptability survey. The quantitative findings suggest that patients generally have a positive attitude toward the analyzed items, particularly Terabot’s clarity of communication, friendly appearance, and the usefulness of its therapeutic exercises, whereas scores for empathy and emotional understanding were only moderate. The qualitative analysis revealed three main areas of patient experience: (1) the therapeutic relationship with Terabot, including both perceived strengths and clear limitations in relational depth, (2) the perceived usefulness of therapeutic exercises, especially relaxation techniques, and (3) Terabot’s physical features, such as its appearance and voice. Patients highlighted contemporary nature of the tool, as well as its limitations, including technical issues (such as freezing and stuttering), rigid communication, insufficient personalization of exercises, and a lack of empathy. Facilitators’ observations confirmed these limitations while also noting patient engagement and openness to technological innovation.

**Conclusion:**

This preliminary study suggests that Terabot may be an acceptable and potentially useful tool for supporting therapeutic work with patients with schizophrenia. However, the findings must be interpreted with caution, given the study’s exploratory design, the time-limited therapeutic intervention, and technical limitations. Enhancements in relational responsiveness, dialogic flexibility, and the range of therapeutic exercises are necessary before drawing more definitive conclusions. Moreover, controlled clinical trials are required to evaluate Terabot’s effectiveness and its capacity to foster a stronger therapeutic alliance.

## Introduction

Schizophrenia is a chronic mental disorder characterized by the presence of at least two domains (from among delusions, hallucinations, disorganized speech, behavior, and negative symptoms). According to ICD-11, in order for a diagnosis of schizophrenia to be made, the symptoms should persist for a minimum of one month plus allowance for residual phases [1]. Therapy is recommended in the management of patients; cognitive-behavioral therapy for psychosis occupies a special place in guidelines such as those of the WHO and NICE [2]. In recent years, the use of AI in mental healthcare, including psychiatry and psychotherapy, has become an increasingly popular topic [3,4]. Machine learning constitutes a crucial foundation of contemporary AI systems, including those applied in mental healthcare [5]. Such systems offer several advantages that are particularly relevant for the development of therapeutic applications. They can adapt and make self-corrections, thereby better handling unstructured data, which is particularly relevant in therapeutic processes [6,7]. Complex machine learning algorithms have been employed in studies on innovative tools, designed to predict treatment outcomes in individuals with schizophrenia [8] and to develop tools for enhancing psychiatric diagnostics [9]. The application of artificial intelligence in psychotherapy has also been increasingly well described. AI chatbots have been shown to contribute significantly to reducing suicidal, depressive, and anxious thoughts [10,11]. A meta-analysis confirmed the short-term effectiveness of these interventions compared with control conditions, showing statistically significant effects in the following areas: reduction of depressive symptoms, generalized anxiety, specific anxiety symptoms, improved quality of life and well-being, stress reduction, and psychosomatic disorders [12]. A particularly interesting direction of research is the use of artificial intelligence in therapy for people with schizophrenia. Recent developments in technology-assisted interventions for schizophrenia can be broadly classified into three therapeutic types: (1) text-based chatbots, (2) immersive VR-based therapies, and (3) embodied conversational AI agents. Text-driven chatbots are designed to deliver psychological support through writing-based, automated dialogue systems. They have shown preliminary potential for enhancing access to care and support symptom monitoring, motivation, and aspects of cognitive functioning; however, evidence remains early-stage and should be interpreted with caution, particularly in populations with schizophrenia. Examples include the AI-driven mobile intervention PRIME, designed to enhance reward processing and motivation in individuals with recent-onset schizophrenia spectrum disorders, which demonstrated feasibility in a remote randomized controlled trial [13]. Similarly, SchizoBot, a chatbot employing an artificial neural network to deliver cognitive-behavioral strategies, illustrates how conversational systems can be tailored for individuals with schizophrenia [14]. Broader mHealth interventions within this domain address adherence to medication, cognitive training, and symptom management, but the current evidence remains limited by small sample sizes and the scarcity of randomized controlled trials [15]. A scoping review has further highlighted the growing role of chatbots in rehabilitation, including applications in symptom monitoring, pharmacotherapy management, relapse prediction, functional training, and psychosocial support, while also noting the scarcity of randomized controlled trials in this area [16,17]. The second category involves virtual reality (VR)-based interventions, which leverage a high degree of sensory immersion to create environments perceived as real, thereby enabling controlled exposure to scenarios associated with threat or distress. Recent clinical trials have demonstrated that VR-based cognitive therapy, such as the gameChange program, can significantly reduce agoraphobic avoidance, distress in everyday situations, and ideas of reference and persecutory delusions, among individuals with schizophrenia spectrum disorders, especially those with high levels of anxiety and avoidance [18]. VR platforms allow patients to gradually confront feared social environments in a controlled, reproducible manner, enabling therapists to tailor the intensity of exposure and monitor real-time responses. Additionally, VR-based social cognition training programs have shown promising effects on emotion recognition, theory of mind, and functional outcomes, complementing traditional psychosocial interventions. Despite these advances, more research with long-term follow-up and larger samples is required to establish the durability of therapeutic gains and identify moderators of treatment response. Evidence indicates that VR interventions show moderate effectiveness in reducing delusional symptoms and may outperform traditional therapeutic approaches, although further research is needed to clarify underlying mechanisms and establish long-term efficacy [19,20]. The third emerging approach centers on embodied conversational agent**s**, that is, interactive systems equipped with a visual or auditory embodiment designed to engage patients in realistic, relationally meaningful dialogues, an approach exemplified by interventions such as Avatar Therapy [21]. Embodied relational agents, comprises avatar-based and agent-based psychotherapeutic interventions focused on relational and dialogical mechanisms of change. Initiated by Julian Leff, Avatar Therapy enables patients to engage in structured dialogue with a digital representation of their distressing voice, facilitating emotional processing, and a renewed sense of control. Subsequent studies have shown significant reductions in the frequency and distress of auditory hallucinations, improved functioning, and meaningful therapeutic engagement [22–24]. Related approaches, including emerging embodied agents such as Terabot, build on similar principles by offering human-like interactions designed to support emotional expression, relational work, and therapeutic dialogue. From a conceptual standpoint, Terabot aligns most closely with the embodied conversational agent paradigm, as it combines natural-language interaction with an artificial persona intended to support emotional regulation and relational engagement. This places it within the same broader family of technologies that aim not merely to simulate conversation but to recreate aspects of interpersonal exchange relevant to psychotherapeutic processes. The distinction between the three technology-assisted therapeutic approaches used among patients diagnosed with schizophrenia: chatbots, immersive VR-based therapies, and embodied conversational agents, is significant. Each of these tools targets different mechanisms of change and serves distinct therapeutic functions.

The literature also reports several potential adverse effects of artificial intelligence – based interventions among individuals diagnosed with schizophrenia, including misunderstandings of AI-generated content, reinforcement of maladaptive beliefs, and overstimulation resulting from technologically rich environments [25]. These findings underscore the fact that the integration of AI into therapeutic processes for people with psychosis is associated not only with promise but also with a degree of clinical uncertainty. Consequently, the development and implementation of such technologies evoke both hope, regarding improved access, personalization, and scalability of care, and concern about unintended psychological or behavioral consequences. Given this dual landscape, a systematic investigation into the efficacy, safety, and acceptability of these innovative treatment modalities is essential. This includes not only controlled clinical trials assessing therapeutic outcomes but also qualitative research elucidating patient experiences, perceived benefits, and barriers to engagement. Such analyses are vital for identifying the full therapeutic potential of AI-assisted interventions, clarifying their limitations, and informing evidence-based guidelines to ensure their responsible and clinically appropriate use.

It has been observed that despite the substantial increase in research and efforts to evaluate the effectiveness of technologically supported therapeutic methods, there is still an insufficient number of studies examining the use of these methods in groups of patients with severe mental disorders, such as schizophrenia [17].

### Emotions in patients with schizophrenia spectrum disorders

Dysfunction in emotional regulation is an important clinical issue in patients with schizophrenia [26]. Gross’s concept of emotions emphasizes that they are dynamic, with regulatory processes playing a key role in their course. Gross distinguishes three essential stages of emotion regulation: situation selection, identification of the need for regulation, and implementation of strategies. Previous research evidence indicates that schizophrenia is associated with abnormalities at each of these stages of emotion regulation [27,28].

Based on Gross’s concept, we analyzed the difficulties experienced by patients with schizophrenia in three stages. It was found that patients with schizophrenia exhibited an ineffective threshold for identifying the need for regulation—they attempted regulation too frequently at low levels of negative affect and too infrequently at high levels, while investing inadequate effort in regulation [29]. It was noted that these patients had a limited repertoire of regulatory strategies and a tendency to choose maladaptive ones [30]. It was further indicated that individuals with schizophrenia demonstrated an impaired implementation of many regulatory strategies, likely resulting from cognitive and neural dysfunctions. This manifested, among other things, in excessive switching between strategies and delayed disengagement compared to control groups [31]. These data became the basis for developing short therapeutic exercises implemented within Terabot. Their design was based on cognitive-behavioral therapy (CBT), one of the most thoroughly researched and widely used methods for the treatment of mental disorders, including schizophrenia [32,33]. Clinical inspiration was also drawn from the so-called “third wave of CBT,” which includes approaches such as Acceptance and Commitment Therapy (ACT), Dialectical Behavior Therapy (DBT), and Mindfulness-Based Cognitive Therapy (MBCT). These approaches emphasize the acceptance of emotional experiences, psychological flexibility, and mindful, conscious responding instead of automatic and maladaptive strategies [34–37]. A direct conceptual foundation was Barlow’s transdiagnostic protocol – the Unified Protocol for Transdiagnostic Treatment of Emotional Disorders – which assumes that many mental disorders, from anxiety and depression to psychotic disorders, share common mechanisms underlying difficulties in emotion regulation [38].

In this study, we assessed the applicability of Terabot virtual therapists in brief interventions aimed at emotion regulation in individuals with schizophrenia.

The primary aim of the study is to evaluate the acceptability of the intervention based on an embodied conversational agent (Terabot) in individuals diagnosed with schizophrenia.

The additional (exploratory) aims are:

1. To assess the feasibility of the intervention, including implementability and potential technological barriers.
2. To analyze relational dynamics between the participant and the conversational agent, with particular emphasis on the quality of the dialogue.
3. To identify potential limitations and risks associated with the use of AI-supported interventions in patients with severe mental disorders.

## Methods

### Study design

The study was designed as a mixed-methods investigation, combining quantitative and qualitative approaches to obtain a more comprehensive picture of patients’ experiences in therapy with Terabot. The qualitative appraisal of the study was conducted with reference to the COREQ criteria (Consolidated Criteria for Reporting Qualitative Research) [39], with a full report provided in S1 Table. The reporting standard was applied consistently, both in the preparatory phase (focus group and development of the therapeutic protocol) and in the active research phase (implementation of the intervention).

To characterize the study group, descriptive statistical analysis was conducted, enabling a concise and transparent presentation of key sociodemographic and clinical features. Statistical analyses were conducted using IBM SPSS Statistics 30. Basic descriptive statistics, along with the Shapiro–Wilk test and the Student’s t-test for dependent samples, were performed. The level of significance in this chapter was set at α = 0.05. In parallel, a qualitative thematic analysis was performed on patients’ survey responses and facilitators’ observational notes, allowing an in-depth exploration of participants’ experiences and perceptions of the intervention.

### Criteria for inclusion

The study was approved on 27 April 2022 by the Ethics Committee of the Institute of Psychology and Neurology in Warsaw, Poland; resolution No. IV/2022, and it conforms to the provisions of the Declaration of Helsinki. The research was conducted between April 2022 and August 2023. Participants were recruited from the Psychosis Relapse Prevention Ward at the Warsaw Institute of Psychiatry and Neurology. The ward admits inpatients with severe mental disorders who are stabilized and in partial or complete remission, with the primary objective of preventing further mental health crises.

The ward offers a comprehensive therapeutic program that includes community meetings, psychoeducation sessions, therapy groups, metacognitive training, initiative workshops, physical activities, and cognitive training. Within this therapeutic environment, patients who additionally met the study’s inclusion criteria were invited to participate in supplementary sessions with the virtual therapist (Terabot).

Eligibility for the Terabot intervention was restricted to patients with diagnoses falling under ICD-10 codes F20.0–F20.9, confirmed by experienced psychiatrists prior to hospital admission [40]. The detailed inclusion and exclusion criteria are provided in Table 1. All participants received thorough information regarding the aim, duration, participation requirements, and potential benefits of the study, accompanied by a full confidentiality clause. Personal data were anonymized.

**Table 1:** Eligibility criteria for research.

Inclusion criteria	Exclusion criteria
• age between 18 and 65, • meets the diagnostic criteria for schizophrenia according to ICD-10 (F 20.0–20.9), • no changes in prescribed pharmacotherapy in the week preceding the study and during the intervention, • no documented mental disability or organic changes of the central nervous system, • stability of mental state adequate for the use of the dialogue agent, • no coexisting active addiction to psychoactive substances, • computer literacy.	• aged under 18 or over 65, • refusal to participate in the study, • acute psychotic crisis, • the use of sedative medications the week preceding the study and during the intervention, • cognitive deficit diagnosed.

### Preparatory phase

Interest in the issue of emotion regulation in patients with schizophrenia dates back to the 1990s, while the last two decades have seen intensive development of social skills and cognitive training, of which emotion regulation was an important component. Increasingly, studies indicate that improving emotion regulation can contribute to a reduction in negative symptoms and improved social functioning [41]. In the rehabilitation ward where the study was conducted, emotion regulation training appeared as part of broader therapeutic programs; there had been no separate module focused solely on this issue [42]. These observations became the inspiration for developing Terabot – a tool designed to support patients in the independent training of emotional regulation [43]. The therapeutic program was based on James Gross’s concept and included elements of psychoeducation, emotion recognition, regulatory exercises (e.g., relaxation and breathing techniques), reframing emotional meanings, and individual tasks to consolidate new ways of coping with emotional experiences.

#### Therapeutic protocol.

During the preparation phase, a focus group was conducted to identify the emotions that would subsequently be targeted in therapeutic work. The meeting of the group took place in a psychiatric ward among patients diagnosed with schizophrenia (according to ICD-10: F20.0–F20.9), in the same ward where the study was conducted. It was a one-time meeting led by two clinicians. One of the facilitators of the session was a clinician (this individual had extensive clinical experience as a psychiatrist, psychotherapist, and supervisor), and the other person (with a background in psychology) had an assisting role. The meeting lasted two hours. The first step involved providing a short discussion on emotions. They were subsequently asked to discuss the emotions they experienced most frequently and the strategies they used to cope with them. Patients’ statements were recorded by the assisting clinician, who did not influence their responses. The therapeutic team then evaluated the collected material to determine which emotions posed the greatest challenges for patients. A thematic analysis of the notes was carried out, leading to the selection of three emotions for the development of therapeutic dialogues and exercises. The group consisted of 10 inpatients.

#### Design and development of a polish-language therapeutic dialogue system.

An initial motivation for creating Terabot was the need to reduce the workload of therapeutic staff in rehabilitation wards, where professionals face significant demands. Terabot was intended to serve as an independent tool that patients could use at any time during their stay, allowing for the systematic development of emotion regulation skills. The first phase included creating a virtual therapist to conduct independent patient conversations. It was a spoken dialogue system for the Polish language based on the RASA platform [44]. The system has been initiated with sample dialogues and then trained during the so-called conversation-driven development. A simultaneous process was the development of the therapeutic protocol. Once the preliminary version of Terabot and the therapeutic protocol were developed, the next step involved conducting a preliminary examination test with a non-clinical group. This stage aimed to improve Terabot’s communication skills and teach him to react to as many responses as possible. After completing this stage, the clinical phase was initiated.

#### Description of the role of facilitators during the sessions with Terabot.

Two facilitators were trained in their tasks for the study. Neither of them had clinical experience. One of them was always present during the entirety of each meeting, supporting the patient in difficulties that might arise during sessions with Terabot. The facilitator’s tasks included leading the patient into the room, assisting during subsequent parts of the session (e.g., prompting the patient to speak more loudly or clearly), and systematically observing and documenting any technical difficulties, emotional reactions, or potential adverse events arising during the meetings with Terabot, including signs of distress, symptom exacerbation, or behavioral changes. Facilitators also explained the content of the individual assignments to the patients. They did not know the participants beforehand and ensured that their responses remained unbiased. The participants were informed about the purpose of the study and their right to withdraw at any time. Each participant consented to the presence of the facilitator. The facilitator took notes on the course of the sessions and described the difficulties that arose during the meetings. Importantly, the facilitator did not provide any therapeutic, emotional, or clinical support and did not intervene in the therapeutic process; their role was strictly limited to technical, organizational, and observational tasks.

## Terabot as a virtual therapist

**Fig 1:**
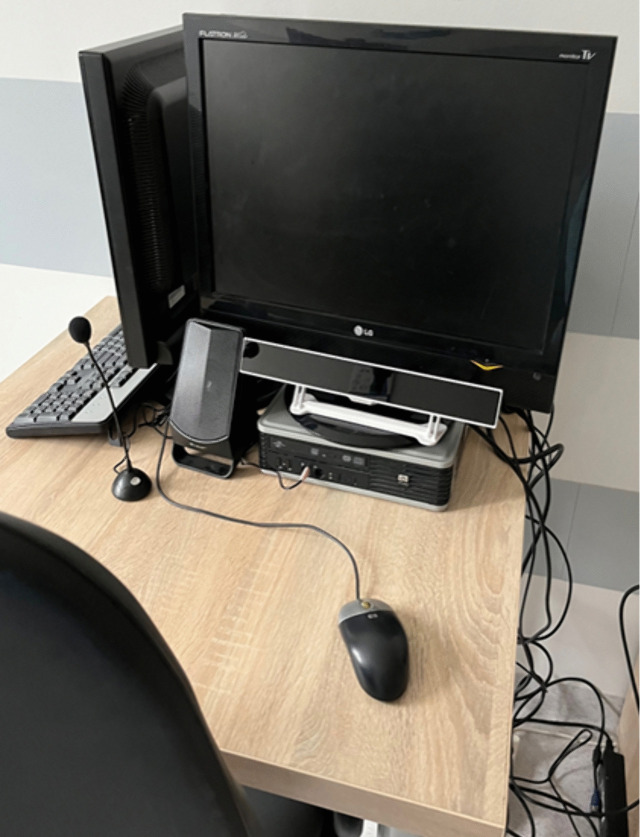
The therapeutic station for patient-Terabot conversations.

Terabot was a task-oriented spoken dialogue system for the Polish language, developed in Poland at the Warsaw University of Technology [45–47]. By offering therapeutic spoken interaction in the Polish language, Terabot contributes to filling a gap in existing dialogue-system research. At the hospital, the therapeutic station consisted of a computer with a screen, a microphone, and speakers. A photograph of this station is shown (Fig 1).

**Fig 2:**
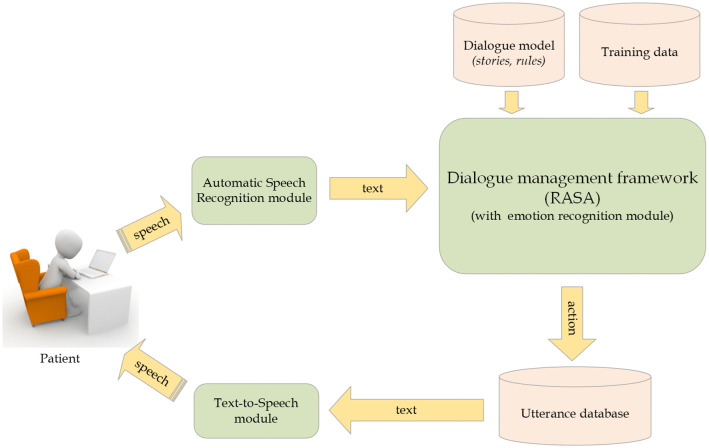
A block diagram of the dialogue system’s architecture.

The mouse was used only to turn on the operating system and the dialogue system. For conducting the conversations, it was hidden on the left side (where the assistant was sitting), being invisible to the patient. The system was equipped with a speech synthesizer and speech recognition system and was therefore capable of communicating with the patients verbally. The architecture of Terabot is shown (Fig 2).

Terabot recognized the patient’s utterances (converted speech into text), interpreted them using the dialogue system engine, and returned an answer in the form of a text compliant with the therapy exercise scenarios provided earlier. A speech synthesizer then converted the answer into speech and played it through the speakers. A computer dialogue system automatically processed the patients’ statements in the following manner:

- intent analysis: the system recognized the intentions behind the patient’s utterance (e.g., recounting an event, sharing emotions, confirming/negating a statement) using the DIET Classifier algorithm [48],
- keyword analysis: detecting and identifying the type of exercise to be performed.

Based on its content, this dialogue system automatically recognized the tone (sentiment) of the patient’s statement (neutral; positive, e.g., happiness; negative, e.g., anger, anxiety); this is done by an algorithm employing an artificial neural network [49,50].

### Physical features of Terabot

**Fig 3:**
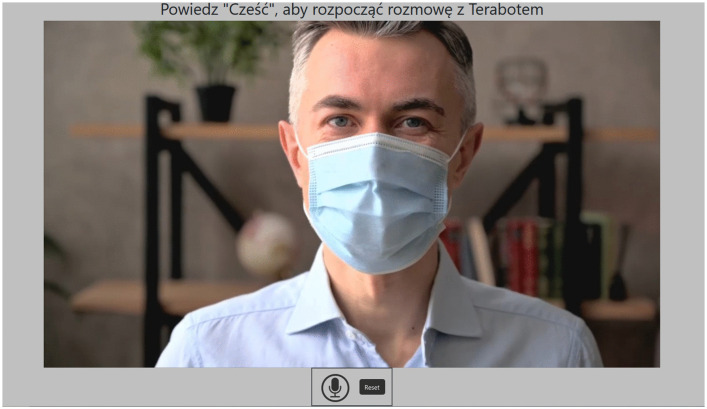
The visual appearance of Terabot in the form of a film of a man wearing a hygiene mask.

While designing Terabot, efforts were made to ensure its appearance was neutral to avoid triggering patients’ biases [51], the appearance is shown (Fig 3). He moved slightly, and his eyes blinked naturally. This gave the patient the impression of sitting in front of the computer during an online session with a therapist. We present its main physical characteristics in Table 2.

**Table 2:** Physical features of Terabot.

Physical Feature	Description
Appearance	Terabot is designed to have a visually appealing and user-friendly interface. The screen displayed a looped movie of a male actor wearing a blue shirt, with the face covered with a hygiene mask. Terabot’s face was slim and pleasant. His face was mobile and active, particularly in the eye area.
Display	Features a high-resolution screen that provides clear and sharp visual interactions.
Sound	Includes a high-quality speaker system and a microphone for natural and intelligible voice communication. The voice of the Terabot is calm and friendly.

Translation from the Polish language: Say “hello” to start a conversation with Terabot.

### Description of the intervention using Terabot

Terabot proposed exercises aimed at conducting a conversation about emotions, increasing awareness and improving the ability to recognize them, and then performing an exercise designed to reduce the intensity of emotion. All the commands and tasks were guided by Terabot. The whole intervention was planned to last five days. During this time the patient conversed with the dialogue agent and participated exercises to improve their ability to deal with emotions. The meetings lasted approximately 20–30 minutes. After completing the exercise, the patient was given an assignment related to the issue discussed in the meeting (personal task).

This dialogue system was prepared (taught) to conduct three therapeutic exercises focused on management of anger, anxiety and shame. The emotions included in the therapy protocols were selected based on a demand analysis conducted during a focus group. The exercises were developed on the basis of principles of cognitive-behavioral therapy and mindfulness-based therapy (Mindfulness Based Cognitive Therapy, MBCT) [52].

Exercises for each of these emotions are built on the same principles:

1. Greeting.
2. Terabot asks if the patient agrees to participate in the exercise.
3. Introduction – specifying which emotion the conversation will refer to.
4. Asking the patient to recall a situation in which they felt the specific emotion.
5. Psychoeducation on the topic of emotion, including its physiological aspects and impact on behavior.
6. Proposing a short exercise aimed at helping the patient cope with a particular emotion.
7. Personal task assignment.

Terabot was also able to handle cases out of the main scenario. If the patient avoided answering the question, e.g., “Do you recall a situation where you felt angry?”, the system offered pre-defined examples, such as “Imagine you come home hungry and realize you forgot to do the shopping.” The patient was allowed to quit the conversation at any point by expressing such a wish. In such a case, the system suggested meeting at another time and said goodbye. Terabot was also able to answer basic questions about the system itself, such as “Who are you?” “Which languages do you speak?”, “Which topics can you discuss?” Following the psychoeducation part on the topic of emotion, the system offered patients a relaxation exercise. It consisted of several relaxation instructions, interleaved with silence. Afterwards, Terabot asked the patients how they felt. If the patients felt well, Terabot thanked them and said goodbye. If they answered they had problems with relaxation, Terabot asked for details and encouraged them to join another session with Terabot.

Table 3 presents the structure of the Terabot intervention, psychotherapeutic stages, and its corresponding stages of emotion regulation according to Gross’s model. The individual dialogue components operationalize, in sequence: the following antecedent-focused strategies (identifying the emotion and its triggering situation), attentional deployment and cognitive reappraisal (psychoeducation, analysis of physiological reactions, assigning new meaning to emotions), as well as response modulation through relaxation and mindfulness exercises. The intervention concludes with an evaluation of the effectiveness of the emotion regulation process and a homework assignment aimed at consolidating and generalizing the acquired skills.

**Table 3:** Terabot operationalizes Gross’s emotion regulation strategies.

Terabot intervention	Description of therapy stage	Terabot operationalizes Gross’s emotion regulation strategies
Good morning! Excellent, welcome! My name is Terabot, I’m helping with your therapy. What exercises shall we do today?	The general introduction refers to the three emotions in an interactive conversation with the patient. Engagement in the therapeutic process	
Today, I would like to work with you on [emotion] • Is it okay if we talk? • Identifying the emotion that is [emotion] is very important. If you learn to do that, it will be easier for you to control [emotion]		Antecedent-focused strategies: a) emotion identification b) situation selection c) linking experienced emotions with a specific situation
• Can you remember a situation in which you felt [emotion]? • Maybe something happened at home, at work, or on the street. Can you remember a situation like that? • Where did it take place?	Identification of the emotion-triggering situation	
• Sometimes you can get angry at another household member for leaving dirty dishes in the sink	Psychoeducation: Terabot provides examples of situations in which the emotion being discussed can appear. In the following example, it is anger.	Cognitive reappraisal: a) psychoeducation about emotions
• Notice how [emotion, here: anger] escalates quickly. Your focus narrows, you concentrate on what makes you angry and ignore other things that might normally interest you. For example, if we’re angry with our partner, we sometimes forget how much we love them. Can you recall the feeling you get in your body when you are angry? How does your body react?		Attentional deployment and cognitive reappraisal – reframing. Directing attention to the emotion and its physiological consequences, as well as assigning new meaning to the experienced emotions. Analysis of the physiological reactions typical for a given emotion. The beginning of an intervention aimed at shifting the response pattern.
• Sweating, increased heart rate, and muscle tension are common symptoms of agitation. How does experiencing them impact your behavior? - What does anger do to you? - What would you do if anger took over you?	Psychoeducation-continuation.	Attentional deployment and cognitive reappraisal – reframing – continuation.
• Unfortunately, that is how anger usually works unless you pay attention to it and try to work on it. - How do you deal with anger? -Do you avoid expressing it, or do you blow up?	Discussion of ways of dealing with the emotion.	Indicating various possible emotion-regulation strategies and initiating change in their use.
I have a proposition for you: We will now perform an exercise allowing you to learn how to decrease tension and anger. Make yourself comfortable, relax, and close your eyes. Experience the place you’re in. Feel the pressure of your body on the chair, on the floor, and feel the space around you. Focus on one sensation, perhaps on the feeling of warmth. Remain within this sensation for a moment. How do you feel? Are there any emotions other than anger that you’re feeling? Do you feel peaceful?	Relaxation/mindfulness exercises	Initiating an exercise aimed at reducing the intensity of experienced emotions, assigning them new meaning, and reframing the meaning of the situation that triggered the emotions (response modulation). Attentional deployment: Mindfulness/ Redirecting attention (changing the focus of attention – reducing rumination and the intensity of anger). Reappraisal (meta-cognitive evaluation of the emotional state).
If so, try to visualize it. Feel it encompasses you and remain within that feeling for a moment. Are you less angry now?		Monitoring and evaluation of the effectiveness of the emotion regulation skills developed
Homework: Repeat this exercise daily. I will be happy to help.	Personal assignment	Regulation Strategy Maintenance

### Scales and survey used in the study

Sociodemographic data were collected using a questionnaire prepared by the research team, which included the following information: age, gender, education, marital status, employment status, and place of residence. Treatment-related information collected included diagnosis, number of hospitalizations, and current treatment. An acceptability survey was prepared to evaluate the use of a conversational agent, which patients completed after their interactions with Terabot. The Davis technique was used to assess the validity of the items developed [53]. The questions were assessed by four independent experts, comprising two clinicians and two technology specialists. Each question was rated on a scale from 1 to 3 (1 – not relevant, 2 – useful but not essential, 3 – essential). Calculations were performed for each question, and only items with a content validity index > 0.80 were included in the final survey. The internal consistency of the acceptability survey (Q1-Q12) in the present sample was Cronbach’s α = 0.84. Below are the questions used to measure the acceptability of the use of the dialogue agent:

Q1. The statements made by Terabot were understandable to me.
Q2. I felt that Terabot understood what I wanted to say.
Q3. I felt that Terabot understood my emotions.
Q4. The questions asked by Terabot were appropriate.
Q5. The exercises proposed by Terabot were helpful.
Q6. The statements made by Terabot sounded natural.
Q7. Terabot spoke too fast for me.
Q8. I enjoy talking with Terabot.
Q9. The presence of the observer was helpful to me.
Q10. Terabot looks friendly.
Q11. I feel that therapy with Terabot helped me.
Q12. Computer-based methods, such as Terabot, can be used in therapy.

Patients related to the above statements on a 5-point Likert Scale (1 - totally disagree, 2 - disagree, 3 - hard to say, 4 - agree, 5 - totally agree).

At the end of the survey, we included open-ended questions:

- What did you like the most about the exercise with Terabot?
- What did you like the least about the exercise with Terabot?
- If you wish, you can add your additional comment.

The overall severity of psychopathological symptoms was measured with the standard version of the BPRS (Brief Psychiatric Rating Scale) [54]. The BPRS is one of the most widely used scales for the measurement of psychopathologies. It includes 18 symptoms assessed by a clinician on a 7-point scale (1 = symptom not present; 7 = symptom extremely severe) (Cronbach’s *α* = 0.72). An additional element of the qualitative assessment consisted of notes taken by the facilitator and presented during each session in which the patient interacted with Terabot. There were three types of issues observed by the facilitators:

- Technical Issues and Functionality
- Quality and Personalization of Interaction
- Facilitator’s presence during sessions with Terabot.

### Safety monitoring and adverse events

Adverse events were operationally defined as any unintended deterioration in participants’ mental state, including: (1) clinically significant exacerbation of psychotic symptoms, (2) emergence or disclosure of suicidal ideation and an associated increase in suicide risk, and (3) heightened levels of anxiety and psychomotor agitation. Safety monitoring was conducted indirectly through the administration of the Brief Psychiatric Rating Scale (BPRS) at baseline and following completion of the intervention. Additionally, qualitative safety monitoring was supported by systematic observations recorded by a facilitator present during each session. The facilitator documented participants’ behavioral and emotional responses throughout the meetings. In cases where potential adverse events were identified, the facilitator was authorized to notify the patient’s treating psychiatrist regarding any observed deterioration in mental state, thereby enabling appropriate clinical follow-up.

## Results

### Socio-demographic data

The study group comprised 35 patients (20 men and 15 women). The mean age of respondents was 37.1 years (SD = 11.1), ranging from 22 to 64 years. The average number of hospitalizations was 6.1 (SD = 5.0), with values ranging from 1 to 20. All patients had F20.0 - F20.9 diagnoses according to ICD-10 (according to ICD-10: F20.0). All patients were being treated with antipsychotic medications. Table 4 below shows descriptive statistics for age and number of hospitalizations.

**Table 4:** Summary table – Descriptive statistics of the characteristics of the group.

Variable	Mean (M)	Standard Deviation (SD)	Median	Minimum	Maximum
**Age**	37.14	11.11	35.0	22.0	64.0
**Hospitalizations**	6.09	5.03	4.0	1.0	20.0

M – mean; SD – standard deviation.

Table 5 below presents the basic sociodemographic data of the respondents. The majority of the participants were male (57.1%) and single (77.1%). More than half were retired or on disability pension (51.4%). The largest proportion of participants (31.4%) reported residing in cities with populations exceeding 100,000. The majority of patients had either secondary (51.4%) or higher (31.4%) education.

**Table 5:** Sociodemographic characteristics of the study group (N = 35).

Variable	N	%
**Gender**		
Male	20	57.1
Female	15	42.9
**Marital status**		
Single	27	77.1
Informal relationship	2	5.7
Divorced	2	5.7
Separated	4	11.4
**Employment status**		
Employed	9	25.7
Unemployed	7	20.0
Student	1	2.9
Retired/Disability pension	18	51.4
**Place of residence**		
Countryside	8	22.9
Town < 50k	10	28.6
City 50–100k	6	17.1
City > 100k	11	31.4
**Education**		
Primary	1	2.9
Vocational	1	2.9
Secondary	18	51.4
Incomplete higher	4	11.4
Higher	11	31.4

n – number of respondents; % – percentage.

### BPRS

This scale was used to monitor and confirm the stability of participants’ mental state during the study. BPRS data were available for 34 participants. One patient did not complete the scale and was therefore excluded from the analysis. The Shapiro–Wilk test did not reveal significant deviations from the normal distribution. The analysis revealed no statistically significant differences in the BPRS scales (BPRS T0: M=2.15; SD=0.51; BPRS T1: M=2.02; SD=0.57; t=1.44; df=33; p=0.16).

### Analysis of the acceptability survey and experiences with Terabot therapy – quantitative and qualitative analyses

**Fig 4:**
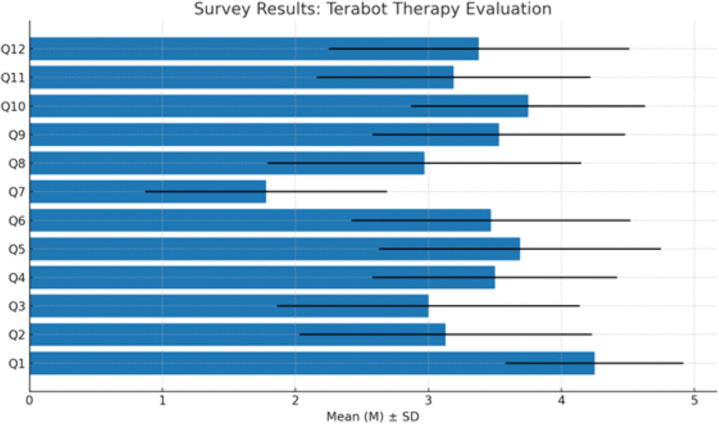
Distribution of mean scores (M) with standard deviations (SD) for each survey question. M – mean, SD – standard deviation, Q1-Q12 – survey questions (1-12) posed to patients.

The assessment was performed by analyzing the responses to closed-ended survey questions, analyzing the open questions in the survey, and the facilitator’s observation. Fig 4 and Table 6 present the patients’ responses to closed–ended questions. We obtained responses from 32 patients participating in the study. Three people refused to complete the survey.

**Table 6:** Descriptive statistics of the closed-ended survey questions.

Questions:	Mean (M)	Median (Me)	Standard Deviation (SD)
**Q1.** The statements made by Terabot were understandable to me.	4.25	4.0	0.67
**Q2.** I felt that Terabot understood what I wanted to say.	3.13	3.0	1.10
**Q3.** I felt that Terabot understood my emotions.	3.0	3.0	1.14
**Q4.** The questions asked by Terabot were appropriate.	3.5	4.0	0.92
**Q5**. The exercises proposed by Terabot were helpful.	3.69	4.0	1.06
**Q6.** The statements made by Terabot sounded natural.	3.47	4.0	1.05
**Q7.** Terabot spoke too fast for me.	1.78	2.0	0.91
**Q8.** I enjoy talking with Terabot.	2.97	3.0	1.18
**Q9**. The presence of the observer was helpful to me.	3.53	4.0	0.95
**Q10**. Terabot looks friendly.	3.75	4.0	0.88
**Q11.** I feel that therapy with Terabot helped me.	3.19	3.5	1.03
**Q12.** Computer-based methods, such as Terabot, can be used in therapy.	3.38	3.0	1.13

Mean (M), Median (Me), and Standard Deviation (SD) for all closed-ended survey questions.

Most questions received neutral and positive ratings – the responses “hard to say” (3) and “agree” (4) were the most dominant. The mean values ranged mainly between 3.1 and 4.3**,** indicating that patients were generally positively inclined, although not unanimous in all aspects. The highest-rated questions (mean approx. 4.2–4.3) show that participants were satisfied with the therapy. Questions with lower averages (approx**.** 3.0–3.1**)** suggest that opinions in these areas were divided – some patients were neutral or dissatisfied. The analysis included one reverse-coded question: **“Terabot spoke too fast for me.”** In this case, a higher score indicated greater agreement with this statement. Standard deviations of 1 or higher indicate considerable variability in opinions on certain questions.

Qualitative analysis was exploratory and based on thematic content analysis of open-ended survey responses and facilitators’ notes. Two researchers independently coded the data, developed categories and themes, and resolved discrepancies through discussion. Researcher triangulation was applied to enhance credibility, and illustrative participant quotations were included in the results. The analysts acknowledged the potential influence of their clinical and research backgrounds, including the fact that one study leader was employed in the ward where the research was conducted. Despite the pilot nature of the study and the limited dataset, recurring patterns were identified, indicating preliminary thematic convergence. The analysis was conducted manually without the use of specialized software. Three overarching themes emerged: (1) physical characteristics of Terabot, (2) relational and communicative aspects of the interaction, and (3) perceived therapeutic usefulness of the exercises [55].

Based on the responses to the open-ended questions (“What did you like most about the exercise with Terabot?”), the following issues have been identified in several areas, see Table 7. We analyzed the responses in three categories. Table 7 shows examples of patients’ statements from each theme and category.

**Table 7:** Responses to the question “What did you like most about the exercise with Terabot?” categorized into three key themes.

Themes and category	Description	Example quotations
**Theme 1.** Physical features of Terabot **Category 1.1** Appearance and presence **Category 1.2** Sensory features	Participants frequently highlighted the Terabot’s appearance and sensory features as particularly appealing. They described Terabot as “natural-looking” and noted that its “calm and pleasant voice” contributed to a sense of comfort. Some respondents appreciated the robot’s moving image and overall presence, indicating a positive emotional reaction to its physical design.	• “What I liked most during the exercises with Terabot was that it looked natural, I felt comfortable in its presence...” • “Terabot’s moving image” • “I liked Terabot itself” • “Pleasant voice and gaze” • “Calm voice”
**Theme 2.** Relationship between Terabot and the patient **Category 2.1** Perceived engagement and responsiveness **Category 2.2** Social experience of interaction	Patients valued the interpersonal quality of the interaction with Terabot. They emphasized feeling listened to, understood, and engaged. For some, the robot offered an opportunity for conversation and objectivity unavailable in typical clinical interactions. Participants also appreciated the novelty of interacting with a robot and the perceived accuracy of its statements.	• “It was interested in my problems” • “The questions formulated and the accuracy of the statements” • “The novelty of working with a robot” • “I could talk to someone” • “Engaging in an interesting discussion” • “What I like most is objectivity during interactions with Terabot” • “It understood emotions” • “The fact that it understood my statements” • “Comfort”
**Theme 3.** Therapeutic usefulness of the exercises **Category 3.1** Relaxation and calming effects. **Category 3.2** Practical therapeutic value	Respondents pointed to the therapeutic benefits of the exercises, especially their relaxing and calming nature. Several participants noted that the proposed tasks could be used in daily life. They also appreciated the innovative and unique approach offered by Terabot.	• “Relaxation exercises” • “Proposed exercises that I can later use in my life” • “A method that induces relaxation” • “The opportunity to try out a new form of therapy” • “Unique approach”

In response to the question about what patients liked most in working with Terabot, they most often emphasized the relationship and communication with the virtual therapist (e.g., the possibility of conversation, being understood, and its calm manner of speaking). The second important aspect was the therapeutic program itself, namely the proposed exercises and relaxation methods. Patients also drew attention to Terabot’s physical features – including its appearance, voice, and way of presenting itself. Among the responses classified as “other”, the most frequently mentioned was the contemporary character of the form of therapy and the opportunity to try something new. When conducting the thematic analysis of responses to the question about what patients liked the least in working with Terabot, we also aimed to distinguish the three categories described. See the following Table 8.

**Table 8:** Responses to the question: “What didn’t you like most about the exercise with Terabot?” categorized into key themes and categories.

Themes and categories	Description	Example quotations
**Theme 1**. Physical features of Terabot **Category 1.1** Facial expression and appearance **Category 1.2** environmental or sensory conditions	Some participants expressed discomfort related to Terabot’s physical or sensory attributes. Comments referred primarily to the robot’s facial expression, perceived as unnatural or inappropriate, as well as technical or environmental aspects such as overly bright lighting during the sessions.	“The impression that Terabot’s face is smiling.” “Too-bright lighting.”
**Theme 2.** Relational and communicative Aspects of the interaction **Category 2.1** Limited understanding and responsiveness **Category 2.2** Lack of conversational depth or variability	Several respondents reported difficulties related to communication with Terabot. They described situations in which the robot did not fully understand their responses, required repetition, or failed to provide adequate follow-up questions. Participants noted a lack of depth in the conversation, the repetitive nature of the robot’s statements, and an overall impression that Terabot moved too quickly to the exercises without exploring patient input.	“It didn’t fully understand my response.” “I had to repeat or say things differently when it didn’t understand.” “The bot’s functionality – simple statements weren’t understood properly.” “Terabot didn’t fully understand me.” “Lack of specific answers/sense in words after my response.” “No follow-up questions.” “Terabot only focused on moving to the exercises, didn’t want to know the details.” “The same statements with every answer.”
**Theme 3.** Therapeutic aspects of the exercise **Category 3.1** Limited relevance or personalization **Category 2.1** Monotony or low engagement	Participants pointed out that some exercises were not well aligned with their individual needs or clinical situations. Others perceived the tasks as repetitive, monotonous or insufficiently engaging. One recurring issue was that Terabot seemed focused on procedural delivery of exercises rather than tailoring content to the patient’s context.	“It’s hard to match materials to the participant-patient’s situation.” “The proposed exercises.” “Terabot only focused on moving to the exercises, didn’t want to know the details.” “Monotony in the exercises.”

The qualitative findings indicate that patients’ experiences with Terabot were shaped above all by the perceived therapeutic relationship. Interestingly, this aspect was mentioned both as the most and the least satisfying element, suggesting that the quality of relational experience with the virtual therapist was central to a patient’s evaluation. The therapeutic exercises and relaxation methods were also valued, though for some participants they represented a source of dissatisfaction. Physical features of Terabot, while less prominent, contributed in both positive and negative ways. Taken together, the results highlight that even in technology-mediated interventions, the relational dimension remains crucial, while content and form play a secondary yet meaningful role. In the survey, patients could also provide their own free comments about the therapy with Terabot, as presented in Table 9 below.

**Table 9:** Grouped open-ended patient comments*.*

Theme	Categories	Description	Example quotations
**1. Perceived Value and Overall Experience**	1.1 General satisfaction 1.2 Positive emotional response 1.3 Perceived usefulness of the exercises	Many participants described their experience as positive, interesting, and satisfactory. They appreciated the innovative nature of Terabot and perceived some of the exercises as effective.	“In general, everything was fine.” “Interesting.” “I am glad that I could take part in the research on this project.” “I’m happy with the Terabot test.” “It was a good conversation overall.” “The final exercises were effective:)” “Terabot is a cool and modern form of therapy.”
**2.Communication Challenges and Emotional Trust Barriers**	2.1 Difficulties with understanding 2.2 Emotional distance and trust issues 2.3 Desire for more advanced interaction	Participants noted difficulties related to mutual understanding and reported challenges in being fully open with a machine. Several expressed that conversations should take place on a higher, more comprehensible level.	“Sometimes Terabot didn’t understand me, when I answered, I had to put it in different words.” “It’s hard to be honest with the machine, you don’t know where it will go..!?” “Someday I hope it will be a conversation on a higher, more understandable level.”
**3. Future Potential and Development Needs**	3.1 Need for more natural appearance 3.2 Need for system improvement 3.3 Repetitive or insufficient exercises	Many participants believe that Terabot has potential, but requires further development, particularly in personalization, emotional understanding, and reducing the repetitiveness of exercises.	“Personally, I think that this type of therapy does not fulfill its role so far. The exercises… were not helpful and seemed repetitive.” “If it develops, it can really be something helpful.” “A Terabot would be better if it had an uncovered face and a natural expression. A computer is not a human being.” “I think it may prove helpful for future generations.” “I recommend it to everyone who wants to work on their own emotions.”

### Qualitative analysis – facilitators’ observations

In the next step, the facilitator’s comments were subjected to thematic analysis. Three main thematic areas were distinguished**:** (a) technical issues and functionality, (b) quality and personalization of interaction, and (c) the role of the facilitator’s presence during sessions:

a) Technical Issues and Functionality:
  - Terabot faced difficulties understanding certain responses, particularly lengthy, unclear, or stereotypical ones.There were issues with low reactiveness, including unrelated or non-empathic responses, lack of emotional engagement, and failure to acknowledge patient input.
  - Errors during tasks necessitated restarts, indicating a lack of task robustness.
  - Terabot struggled to guide patients through recalling emotional memories or facilitating deeper discussions.
b) Quality and Personalization of Interaction
Behavioral and communication barriers:
  - Patients exhibited passive or non-engaged behavior, such as failing to complete tasks, providing short or irrelevant responses, or outright ignoring Terabot.
  - Some patients expressed discomfort or frustration, including speaking quietly, shouting, or showing delayed responses.
  - Cognitive challenges, such as struggling to recall emotional memories or maintaining focus during exercises, impacted interactions.
Emotional and Relational Disconnect:
  - Patients expressed embarrassment or discomfort in communicating with a machine, especially one they perceived as “non-human” or lacking empathy.
  - Some patients expected an “in-depth conversation” but were disappointed by Terabot’s limitations.
  - Patients displayed avoidance behaviors (e.g., looking down, truculence) and skepticism about Terabot’s role.
c) Facilitator’s presence during sessions with Terabot:
  - For most patients, the facilitator’s presence was not bothersome, suggesting that it did not interfere with the interaction.
  - In some cases, however, patients turned to the facilitator for reassurance, reflecting a need for human validation and encouragement.

The analysis of patients’ open-ended responses and the facilitator’s observations indicates that the experiences related to using Terabot were diverse and included both positive elements and limitations in the functionality of this tool. In open-ended responses, patients pointed out its physical features as crucial during the exercises. Some patients confirmed that the physical features of Terabot (e.g., natural manner, voice, movement) enhance patients’ positive experiences and increase their engagement in therapy. Some patients give positive evaluations of the innovation and uniqueness of this type of therapy. The therapeutic exercises were perceived as effective and practical, with a strong focus on relaxation and their potential application in everyday situations. One of the most essential elements that the patients mentioned was the therapeutic relationship with Terabot**.** Among the frequently mentioned aspects were the fluency of this relationship and its deepening. These elements were deemed significant both as aspects appreciated by patients and as areas identified for improvement. Technical issues**,** such as freezing and stuttering, were significant limitations to Terabot’s functionality. Patients also noticed artificiality in its behavior and visual aspects. Patients experienced a lack of fluency in communication, limitations in understanding their responses, and inflexibility in dialogue, which negatively impacted their relationship with Terabot. Some patients found the exercises insufficiently tailored and lacking variety. Limiting interactions to exercises alone was perceived as a lack of engagement and flexibility.

In this final section, we have analyzed the distinct areas in the opinions of patients and the facilitators, as well as those that overlapped. Both patients and the facilitator recognized Terabot’s limitations in communication, empathy, and task execution. Both groups agreed that the tool requires improvements to better meet user needs. Patients placed greater emphasis on Terabot’s potential and highlighted the positive aspects of its operation, while the facilitator focused on a critical analysis of its shortcomings. The patients’ response to the presence of the facilitator was not fully reflected in their feedback.

### Safety monitoring and adverse events

The analysis revealed no statistically significant differences between pre- and post-intervention BPRS scores, suggesting the absence of overall deterioration in participants’ mental state over the short observation period. Qualitative data and conversation transcripts did not indicate the presence of suicidal ideation or a worsening of psychotic symptoms. Facilitator observations showed that some patients experienced episodes of dysphoria and frustration (e.g., raised voice); however, these reactions did not lead to sustained mental health deterioration or necessitate session termination by the facilitator. Consequently, these events were not formally classified as adverse events, which limits the possibility of their quantitative assessment. Overall, the collected data provide preliminary evidence supporting the short-term safety of the intervention; nevertheless, the lack of systematic adverse event monitoring and formal escalation procedures warrants cautious interpretation of the findings.

## Discussion

The present study examined the acceptability of a dialogue-based agent (Terabot) used in brief emotion-focused interventions for patients diagnosed with schizophrenia. Overall, the findings suggest that Terabot was perceived as an acceptable intervention; however, patients also identified several limitations that may constrain its therapeutic usefulness and inform directions for future development. Patient satisfaction emerged as one of the key indicators of Terabot’s acceptability, as reflected in the quantitative results of the acceptability survey. As shown in previous research, patients tend to perceive technology-supported therapeutic interventions, such as those delivered by embodied conversational agents, as modern and attractive forms of treatment [56].

Integrating qualitative and quantitative findings, it becomes evident that communication, the physical features of Terabot, and the nature of the therapeutic exercises played a central role in shaping patients’ experiences. These aspects were consistently highlighted in open-ended responses addressing both positive and negative experiences of interacting with Terabot, and similar observations were reported by the facilitators. Notably, the perceived therapeutic relationship with the virtual therapist appeared to be the most influential dimension. Relational and communicative elements -such as the opportunity for dialogue, a sense of being understood, and Terabot’s calm manner of speaking – were frequently valued by participants.

One of the key findings is the importance of Terabot’s physical features, such as its appearance and the timbre of its voice; the vast majority of patients rated Terabot’s appearance as friendly and its voice as soothing. These results are consistent with studies on the impact of “appearance” and “voice quality” in human – computer interaction, indicating that a neutral and natural appearance combined with an appropriately selected voice can foster a sense of safety and enhance patients’ acceptance of the tool [57].

The uncanny valley phenomenon provides a useful interpretative framework for analyzing patients’ interactions with Terabot [58,59]. Terabot’s visual representation was based on a video recording of a human actor with a partially covered face, a design choice intended to convey neutrality, safety, and professionalism. However, the combination of human facial features with limited facial expressiveness (due to the face mask) may have generated perceptual ambiguity in the studied patient group, making it more difficult to infer the agent’s intentions. Given Terabot’s therapeutic objectives of supporting the emotion regulation process, the limited legibility of the virtual therapist’s emotional cues and communicative intent may have attenuated the clarity of the virtual therapist’s emotional states and intentions, thereby attenuating the expected effects of the intervention [60]. From the perspective of Bordin’s model of the therapeutic alliance, reduced clarity in the agent’s affective signaling may have selectively weakened the emotional bond component of the alliance, despite participants’ generally positive evaluations of the intervention’s structure and of the therapeutic goals and tasks it clearly articulated [61]. Empirical research on the uncanny valley further suggests that experiential discomfort and potential clinical risk are more likely to arise when users construe conversational systems as autonomous relational agents endowed with empathic and mental capacities, while these systems lack the flexibility and affective attunement characteristic of human interaction. Within this framework, attempts to enhance Terabot’s anthropomorphic qualities, although conceptually aligned with efforts to foster relational engagement, may have inadvertently elevated users’ expectations beyond the agent’s actual dialogical and regulatory capacities, resulting in perceived artificiality or emotional distancing in a subset of participants [62].

Although the present study did not employ a dedicated measure of the uncanny valley effect, several of its constitutive elements, including perceived naturalness, affective attunement, and interpersonal comfort, were reflected in both quantitative acceptability indices and qualitative user reports. Collectively, this effectiveness may depend less on maximizing human likeness per se than on ensuring coherence between the agent’s embodied form, communicative behavior, and functional dialogical capabilities, particularly when deployed in clinical populations diagnosed with schizophrenia.

The quantitative findings suggest that Terabot’s main strengths lie in the clarity of its communication, its friendly appearance, an appropriate speaking pace, and the perceived usefulness of some of the therapeutic exercises. Although mentioned less frequently, Terabot’s physical characteristics, such as its appearance, voice, and mode of presentation, also contributed to both positive and negative perceptions. The therapeutic content, including exercises and relaxation techniques, was generally regarded as useful and applicable; however, some participants perceived these elements as insufficiently tailored or lacking variety. These findings are consistent with earlier studies on short digital interventions, where excessive schematization was found to reduce engagement and therapeutic effectiveness [58]. The literature emphasizes that the effectiveness of computer-based conversational agents largely depends on their ability to build relationships with users. In the article [57] the authors define relational agents as computational systems designed to establish and maintain long-term socio-emotional relationships, grounded in mechanisms known from social psychology and interpersonal communication. The authors demonstrate that relational behaviors exhibited by agents, such as empathy, appropriate communication strategies, or the continuation of themes from previous interactions, enhance trust, engagement, and the willingness to continue the interaction. This perspective is particularly relevant in therapeutic interventions, where the quality of the relationship (working alliance) constitutes one of the key predictors of therapeutic outcomes. The significance of the therapeutic relationship in interactions with conversational agents has also been confirmed in prior research on the working alliance in digital therapy. It has been demonstrated that the quality of the relationship with a virtual agent directly influences patients’ engagement, satisfaction with therapy, and adherence to recommendations [57]. Similar conclusions were drawn in a scoping review of embodied conversational agents in clinical psychology [56]. More broadly, it has been argued that the therapeutic relationship remains one of the most powerful predictors of treatment outcomes, regardless of modality, and is equally relevant in digital contexts. In the case of schizophrenia, these findings are particularly meaningful, as impairments in interpersonal relationships are among the core difficulties faced by this patient group [59]. Thus, the possibility of establishing a satisfactory and “safe” therapeutic relationship with a virtual therapist may serve not only as an element supporting therapy but also as a therapeutic goal in itself.

Empathy is closely linked to the capacity for affective attunement, that is, the ability to respond precisely to the emotional tone of the patient’s communication [63]. The rigid, formulaic responses generated by Terabot make such attunement difficult, potentially weakening the patient’s sense of being genuinely understood [64]. These communicative limitations may also lead to disruptions in the therapeutic alliance, as a lack of flexibility and responsiveness is often interpreted as a sign of poor fit, disengagement, or insufficient interest [65]. In the context of relational engagement, it is useful to refer to Bordin’s model, which conceptualizes the therapeutic alliance as consisting of three components: agreement on goals, agreement on tasks, and the emotional bond [61]. Contemporary conversational agent research highlights the point that the design of social cues -including qualities of voice qualities, facial expression, embodied behavior, timing, and discourse style – plays a critical role in shaping the user’s perception of rapport and the alliance. Social cues structure how users interpret an agent’s social presence and relational intent; richer cue design is associated with increased feelings of affiliation, trust, and engagement [66,67,68]. This implies that an alliance is not solely an emergent property of the content of dialogue but is co-determined by the designed of human factors: appearance, vocal attributes, responsiveness, and conversational latency.

Terabot performs well in the first two areas due to the clear structure and transparency of its intervention; however, limited emotional attunement and rigid interaction patterns may weaken the relational component, which is essential for fostering safety and trust. Moreover, the absence of follow-up questions, which in natural conversation serve as mechanisms of mirroring and implicit validation, results in a reduced number of signals that typically communicate understanding and acceptance of the patient’s experiences. As a consequence, the interaction becomes rigid, limiting the depth of emotional exploration.

Terabot belongs to the broader class of relational agents but applies relational dynamics within a therapeutic domain distinct from avatar-based and immersive interventions. Whereas avatar therapy is designed for individuals experiencing persistent auditory hallucinations and focuses on externalizing voices and enhancing perceived control, Terabot targets emotion-regulation processes and aims to modify maladaptive coping strategies, positioning it as a more transdiagnostic tool applicable across psychiatric conditions. Unlike clinician-guided avatar therapy or virtual reality–based approaches that rely primarily on sensory immersion and exposure mechanisms, Terabot operates as an autonomous conversational agent capable of sustaining dialogue independently [69–71]. Despite the absence of a human therapist and immersive technologies, patients in the present study highlighted the importance of relational and communicative aspects of interaction, suggesting that meaningful therapeutic engagement can arise through conversational and relational processes alone. Terabot operates through a fundamentally different therapeutic mechanism, grounded in conversational interaction with an embodied conversational agent. The present findings indicate that relational and dialogic processes may play a central role in shaping user experience and acceptability, even in the absence of immersive environments.

shame).

Taken together, these findings suggest that therapeutic conversational agents should be conceptualized not merely as delivery mechanisms for structured interventions, but as relational systems whose alliance-forming capacities depend on the quality, consistency, and multimodality of their social cues. Future work should thus integrate alliance theory from psychotherapy with design frameworks from human – computer interaction to more systematically examine how users form, maintain, or rupture relational bonds with autonomous agents.

### Limitations of the study

One of the key limitations of the study concerns the absence of an objective measure of emotion regulation (ER), which prevents any reliable assessment of change in the targeted emotional processes. Without standardized ER scales, the evaluation relied primarily on subjective self-reports, which introduces the risk of inflated self-evaluation, particularly in interactions involving socially desirable responding or perceived expectations from the system. This methodological constraint limits the interpretability of the observed effects and restricts the ability to draw conclusions about the intervention’s actual impact on emotion regulation capacities. Consequently, the generalizability of the findings is reduced, and the results should be viewed as preliminary indicators of acceptability rather than evidence of therapeutic efficacy. Future studies should therefore incorporate validated emotion regulation measures and multimodal assessment strategies to allow for more robust inferences regarding clinical change. Another limitation concerns the relatively small sample size, which restricts the generalizability of the findings. Furthermore, the study was of a preliminary and exploratory nature, which means that the results should be interpreted with caution. Technical issues, such as system freezes or limited dialogue flexibility, may have led to bias in patient experiences and therefore affected the outcomes of the evaluation. Finally, the short intervention period did not allow for the observation of longer-term effects or sustainability of potential therapeutic benefits.

### Possible future research

The study presented here is one of the first to assess interventions conducted by a virtual therapist in a group of patients diagnosed with schizophrenia spectrum disorders, evaluating a brief intervention aimed at helping patients manage their emotions. The uncanny valley phenomenon may represent an important direction for future research on therapeutic conversational agents in populations diagnosed with schizophrenia. Systematic investigation of how varying levels of conversational agent realism, emotional expressiveness, and perceived autonomy interact with individuals experiencing psychotic disorders is warranted. Research designed in this manner may contribute to the development of therapeutic tools that effectively support clinical interventions while being optimized in terms of safety, user engagement, and the quality of the therapeutic alliance. Future studies should therefore seek to address questions concerning the optimal degree of anthropomorphism and realism, and their respective effects on acceptability, emotion regulation, and the formation of the therapeutic alliance in clinical populations.

Future research should focus on refining both the technical and therapeutic components of the intervention, with particular emphasis on improving Terabot’s communicative flexibility and empathic responsiveness. The qualitative findings highlight the need for greater conversational scaffolding, including guiding questions and prompts that support more elaborated emotional narratives. Incorporating brief preparatory elements, such as emotion recognition tasks, as well as follow-up questions, may facilitate deeper engagement and more meaningful therapeutic interaction. In subsequent steps, a randomized clinical trial would be necessary. Patients appreciate the modernity and potential of Terabot, but point out specific areas for improvement, such as understanding, the personalization of exercises, and the naturalness of interactions. Further development of the project should take these needs into account to increase its effectiveness and acceptance among users.

## Supporting information

S1 Table This is the S1 Table-COREQ. (DOCX)

S2. Table This is the S2 Table Terabot Ex. Survey. (XLSX)
