# Supplementary material for: A conversational agent as a virtual therapist for patients diagnosed with schizophrenia: A preliminary study
Source: PLoS One. 2026 Feb 27;21(2):e0343519. doi: 10.1371/journal.pone.0343519 (PMC12948111; doi:10.1371/journal.pone.0343519)
Supplement: S1 Table — (DOCX) [file pone.0343519.s001.docx]

**Consolidated criteria for reporting qualitative studies (COREQ): 32-item checklist**

| **No. Item** | **Guide questions/description** | **Reported on Page #** |
| --- | --- | --- |
| **Domain 1: Research team and reﬂexivity** |  |  |
| *Personal Characteristics* |  |  |
| 1. Inter viewer/facilitator | Which author/s conducted the inter view or focus group? | Interviews/focus groups facilitated by two clinicians during preparatory phase; main study facilitated by trained facilitators (psychology background, not clinicians). |
| 2. Credentials | What were the researcher’s credentials? E.g. PhD, MD | Lead author: MD, PhD (psychiatrist, psychotherapist, supervisor). Co-authors: researchers in psychology**, IT engineer** |
| 3. Occupation | What was their occupation at the time of the study? | At the time: psychiatrist/psychotherapist; academic researchers at university faculties. |
| 4. Gender | Was the researcher male or female? | Mixed (male and female researchers). |
| 5. Experience and training | What experience or training did the researcher have? | Lead clinician with extensive clinical training and supervision experience; facilitators trained for the study. |
| *Relationship with participants* |  |  |
| 6. Relationship established | Was a relationship established prior to study commencement? | The main researcher and co-facilitator of the preparatory focus group were staff members of the ward. In the intervention phase, sessions were overseen by external facilitators (with a psychology background) who had no prior relationship with the patients. |
| 7. Participant knowledge of the interviewer | What did the participants know about the researcher? e.g. personal goals, reasons for doing the research | Participants were informed about aims, conditions, and rights; participants were informed about the presence of a facilitator during the sessions. Facilitators were external to the ward and unknown to the patients beforehand. |
| 8. Interviewer characteristics | What characteristics were reported about the inter viewer/facilitator? e.g. Bias, assumptions, reasons and interests in the research topic | The main researcher, who was also employed as a clinician in the psychiatric ward where the study was conducted, facilitated the focus group. This dual role may represent a potential source of bias. However, the main intervention study was conducted largely independently of the main researcher. The external facilitators had a psychology background but no clinical role or prior relationship with the patients. They were trained to provide neutral technical and organizational support, without influencing participants’ responses. |

| **Domain 2: study design** |  |  |
| --- | --- | --- |
| *Theoretical framework* |  |  |
| 9. Methodological orientation and Theory | What methodological orientation was stated to underpin the study? e.g. grounded theory, discourse analysis, ethnography, phenomenology, content analysis | Mixed-methods, thematic analysis and quantitative measures; based on COREQ framework. |
| *Participant selection* |  |  |
| 10. Sampling | How were participants selected? e.g. purposive, convenience, consecutive, snowball | Purposive sampling: stabilized inpatients with schizophrenia (ICD-10 F20.0–F20.9). |
| 11. Method of approach | How were participants approached? e.g. face-to-face, telephone, mail, email | Patients were recruited face-to-face in a hospital ward. |
| 12. Sample size | How many participants were in the study? | 35 participants |
| 13. Non-participation | How many people refused to participate or dropped out? Reasons? | Out of 35 eligible participants, one patient refused to complete the clinical scales, and three patients did not complete the closed-ended part of the acceptability survey regarding experiences with Terabot. |
| *Setting* |  |  |
| 14. Setting of data collection | Where was the data collected? e.g. home, clinic, workplace | Psychiatric ward, Institute of Psychiatry and Neurology in Warsaw. |
| 15. Presence of non-participants | Was anyone else present besides the participants and researchers? | Facilitator present during all sessions. |
| 16. Description of sample | What are the important characteristics of the sample? e.g. demographic data, date | Sociodemographic data: mean age 37.1, 20 male/15 female, majority single, varied education and employment. |
| *Data collection* |  |  |
| 17. Interview guide | Were questions, prompts, guides provided by the authors? Was it pilot tested? | Structured survey developed and validated by experts; interview guide (focus group) used. |
| 18. Repeat interviews | Were repeat interviews carried out? If yes, how many? | No repeat interviews; one session per patient (intervention over 5 days). |
| 19. Audio/visual recording | Did the research use audio or visual recording to collect the data? | No audio or video recordings were collected. Patient responses were documented through written survey forms and facilitators’ observational notes. |
| 20. Field notes | Were ﬁeld notes made during and/or after the interview or focus group? | Facilitators took field notes during each session. |
| 21. Duration | What was the duration of the interviews or focus group? | The focus group lasted approximately 2 hours. Intervention sessions with Terabot lasted 20–30 minutes each, over a 5-day protocol. |
| 22. Data saturation | Was data saturation discussed? | Data saturation was not discussed, as this was a preliminary pilot study with a single focus group and a limited patient sample |
| 23. Transcripts returned | Were transcripts returned to participants for comment and/or correction? | The data came from a single focus group (notes) and from patient-completed surveys; therefore, no transcripts were returned. |
| **Domain 3: analysis and ﬁndings** |  |  |
| *Data analysis* |  |  |
| 24. Number of data coders | How many data coders coded the data? | Two independent coders performed thematic analysis. |
| 25. Description of the coding tree | Did the authors provide a description of the coding tree? | Authors described the coding process and presented grouped categories with illustrative quotations (Tables 8–10). However, no formal coding tree diagram was included. |
| 26. Derivation of themes | Were themes identiﬁed in advance or derived from the data? | Themes were derived inductively from patient responses through thematic content analysis. The coding process allowed new categories to emerge from the data, rather than being pre-identified. |
| 27. Software | What software, if applicable, was used to manage the data? | Quantitative analysis in SPSS; qualitative coding manual, not specific software. |
| 28. Participant checking | Did participants provide feedback on the ﬁndings? | No participant checking of findings reported. |
| *Reporting* |  |  |
| 29. Quotations presented | Were participant quotations presented to illustrate the themes/ﬁndings? Was each quotation identiﬁed? e.g. participant number | Patient quotations presented in tables (see Table 8, Table 9, Table 10). |
| 30. Data and ﬁndings consistent | Was there consistency between the data presented and the ﬁndings? | Consistency between findings and quotations is clear. |
| 31. Clarity of major themes | Were major themes clearly presented in the ﬁndings? | Major themes (relationship, therapeutic exercises, physical features) clearly presented. |
| 32. Clarity of minor themes | Is there a description of diverse cases or discussion of minor themes? | Minor themes and diverse responses discussed (e.g., technical issues, modernity, monotony). |
